# Junctional Rhythms After Transcatheter Aortic Valve Implantation: Incidence, Temporal Patterns, and Clinical Outcomes

**DOI:** 10.1016/j.cjco.2026.03.005

**Published:** 2026-03-18

**Authors:** Grégoire Massoullié, Margot Kachkach, Samuel Adelou, Antoine Boudias, Frederic Jean, Pierre-Antoine Catalan, Saer Abu-Alrub, Nicolas Combaret, Bruno Pereira, Géraud Souteyrand, Romain Eschalier

**Affiliations:** a Cardiology Department, CHU Clermont-Ferrand, Clermont-Ferrand, France; b Université Clermont Auvergne, CHU Clermont-Ferrand, CNRS, SIGMA Clermont, Institut Pascal, Clermont-Ferrand, France; c Biostatistics Unit (Clinical Research and Innovation Direction), CHU Clermont-Ferrand, Clermont-Ferrand, France

**Keywords:** transcatheter aortic valve implantation, junctional rhythm, conduction disturbances, permanent pacemaker, electrophysiology, electrocardiography

## Abstract

**Background:**

Conduction disturbances are common after transcatheter aortic valve implantation (TAVI), yet the clinical implications of junctional rhythm (JR) remain poorly characterized. We aimed to assess the incidence, temporal characteristics, and clinical outcomes associated with JR occurring after TAVI.

**Methods:**

We conducted a prospective study including all TAVI patients at Clermont-Ferrand University Hospital between December 2023 and April 2025. Patients who developed JR were identified, and a propensity score was used to match them in a 1:3 ratio with patients without JR.

**Results:**

Among 616 TAVI procedures, JR was identified in 26 patients (4.2%). The mean time to onset was 41 ± 22 hours after TAVI, with a mean junctional rate of 71 ± 12 beats per minute. At baseline, no significant differences were present in clinical, electrocardiographic, or procedural characteristics between patients with vs without JR. The incidence of in-hospital high-grade atrioventricular block did not differ significantly between groups (11.5% vs 10.3%, *P* = 0.48). Permanent pacemaker implantation occurred in 38.5% of JR patients (10 of 26) vs 16.7% of controls (13 of 78; *P* = 0.02). JR was consistently asymptomatic and transient, and its morphology suggested either Hisian or infra-Hisian origin.

**Conclusions:**

JR after TAVI appeared to be an infrequent and transient phenomenon. Although pacemaker implantation was more frequent among JR patients, no excess of documented high-grade atrioventricular block was observed. The higher pacemaker rate appeared to be related primarily to precautionary management decisions. These findings require confirmation in larger multicentre cohorts.

## Central Illustration

**Figure:**
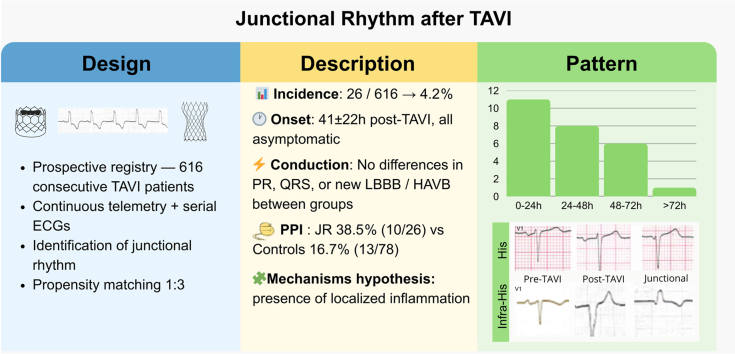

## Résumé

**Contexte:**

Les troubles de la conduction sont fréquents après une implantation valvulaire aortique par cathéter (IVAC), mais les implications cliniques du rythme jonctionnel (RJ) restent mal caractérisées. Notre objectif était d'évaluer l'incidence, les caractéristiques temporelles et les résultats cliniques associés au RJ survenant après une IVAC.

**Méthodologie:**

Nous avons mené une étude prospective incluant tous les patients ayant subi une IVAC au Centre Hospitalier Universitaire de Clermont-Ferrand entre décembre 2023 et avril 2025. Les patients ayant développé un RJ ont été identifiés, et un score de propension a été utilisé pour les apparier selon un rapport de 1:3 avec des patients ne présentant pas de RJ.

**Résultats:**

Sur 616 procédures d'IVAC, un RJ a été identifié chez 26 patients (4,2 %). Le délai moyen de survenue était de 41 ± 22 heures après l'IVAC, avec une fréquence jonctionnelle moyenne de 71 ± 12 battements par minute. Au départ, il n'y avait pas de différences significatives concernant les caractéristiques cliniques, électrocardiographiques ou procédurales entre les patients avec ou sans RJ. L'incidence du bloc auriculo-ventriculaire de haut degré en milieu hospitalier ne différait pas de manière significative entre les groupes (11,5 % vs 10,3 %, *p* = 0,48). L'implantation d'un stimulateur cardiaque permanent a été réalisée chez 38,5 % (10/26) des patients présentant un RJ contre 16,7 % (13/78) des témoins (*p* = 0,02). Le RJ était systématiquement asymptomatique et transitoire, et sa morphologie suggérait une origine hissienne ou infra-hissienne.

**Conclusion:**

Un RJ après IVAC semble être un phénomène rare et transitoire. Bien que l'implantation d'un stimulateur cardiaque ait été plus fréquente chez les patients présentant un RJ, aucun excès de bloc auriculo-ventriculaire de haut degré documenté n'a été observé. Le taux plus élevé d'implantation de stimulateurs cardiaques semblait principalement lié à des décisions de prise en charge par précaution. Ces résultats doivent être confirmés dans des cohortes multicentriques plus importantes.

Since its development, transcatheter aortic valve implantation (TAVI) has profoundly transformed the management of patients with severe aortic stenosis, particularly those at high surgical risk. Despite major technical advances and an overall improvement in procedural safety, conduction and rhythm disturbances remain among the most frequent complications of TAVI, potentially leading to permanent pacemaker implantation (PPI) and prolonged hospitalization duration.^1^, ^2^, ^3^, ^4^, ^5^

Junctional rhythm (JR) remains a poorly explored phenomenon in the context of TAVI. The incidence and clinical significance of JR following TAVI remain largely unknown.^6^ This type of rhythm, more commonly described in other settings such as the postoperative period following cardiac surgery,^7^, ^8^, ^9^ (eg, Fontan procedure), or in association with metabolic imbalances (eg, hyperkalemia or drug-induced pro-arrhythmic effects), may in certain cases reflect injury to or instability of the atrioventricular nodal tissue. In specific situations, the appearance of a JR may precede or accompany the onset of more severe conduction disorders, such as complete atrioventricular block (AVB).^10^

Although some studies have identified this arrhythmia in a non-negligible subset of patients, it has not been the subject of specific recommendations and lacks a defined management strategy.

The present study aims to provide a comprehensive evaluation of JRs occurring in the immediate or early period following TAVI. Specifically, we sought to determine their incidence, electrocardiographic characteristics, and temporal patterns, and to assess their association with the occurrence of advanced conduction disturbances leading to PPI within 30 days after the procedure.

## Materials and Methods

### Study population

Data from patients who all underwent TAVI, between December 1, 2023 and April 1, 2025, at the Clermont-Ferrand University Hospital were collected prospectively. All patients were registered in the JUNCT-TAVI registry (Evaluate the Incidence of Junctional Rhythms Occurring During Post-procedure TAVI Hospitalization, NCT06599008) and provided written informed consent. The study was approved by the local ethics committee (CPP Sud Est VI, 2023/ 24.03051.000665).

### TAVI procedure

The indication for TAVI, the type of valve, and the arterial approach were determined beforehand by a multidisciplinary heart team. The procedures were performed according to the modalities already described.^11^ Clinical events were recorded according to the Valve Academic Research Consortium 3 (VARC 3).^12^

Coronary angiography was routinely performed prior to the TAVI procedure, and patients were revascularized if necessary. All relevant information regarding the clinical and procedural characteristics of the patients was analyzed systematically to address the study objectives. Patient data were analyzed from procedural data as well as from electronic medical records.

### Electrocardiogram (ECG) and assessment of JR

Electrocardiographic parameters and their time of onset were recorded: PR interval (ms), QT interval (ms), QRS morphology, QRS duration (ms), rhythm, and atrioventricular conduction disorders as defined in endpoints. Other relevant ventricular conduction disorders were defined according to the criteria of the American Heart Association, the American College of Cardiology, and the Heart Rhythm Society, as follows: (i) according to morphology: left bundle branch block (LBBB), right bundle branch block (RBBB), nonspecific intraventricular conduction delay (NICD), left fascicular anterior, posterior, and septal; and (ii) QRS interval.^13^ All patients received continuous telemetry for the first 48 hours following the TAVI procedure, and this was extended for an additional 48 hours if an increase in the PR interval or the QRS interval of > 20 ms was observed.

In the case of JR, its heart rate, morphology, and time of onset were recorded. JR was defined as an automatic escape rhythm originating from the atrioventricular junction with reproducible atrioventricular dissociation, QRS intervals that are similar or slightly different from sinus rhythm,^14^ or retrograde P wave, in which atrioventricular conduction could not be identified over several consecutive beats. The clinical impact associated with JR, such as bradycardia (< 50 beats per minute), hypotension (systolic blood pressure < 90 mm Hg), or syncope was recorded when available from the medical charts or telemetry reports. This diagnosis was established either on a standard 12-lead electrocardiogram or through continuous postprocedural telemetry monitoring. A JR was considered to be long-lasting when it persisted for ≥ 24 hours.

All electrocardiographic and telemetry data were reviewed independently by at least 2 experienced cardiologists or electrophysiologists. Discrepancies were resolved by consensus. Continuous telemetry recordings were archived and reviewed daily during hospitalization for arrhythmic events, particularly focusing on JR episodes, atrioventricular conduction status, and heart rate trends.

Electrophysiological studies (EPSs) were performed at the discretion of the treating physician, mostly in patients with JR, as at the time it was suspected to reflect conduction system instability. During the study period, management of post-TAVI conduction disturbances followed institutional practice based on contemporary European Society of Cardiology 2021 pacing guidelines. In patients with new-onset LBBB, significant PR prolongation, or progressive QRS widening, electrophysiological study was frequently performed to assess the HV interval. Pacemaker implantation was considered in cases of documented high-grade AVB or when the HV interval was ≥ 70 ms.

### Endpoints

The primary endpoint was PPI implantation between day 0 and day 30 after TAVI. The secondary endpoint was the occurrence of a high-grade atrioventricular conduction disorder during post-TAVI follow-up as defined by the following: persistent third-degree AVB (cAVB), type 2 AVB (AVB2), and alternating bundle branch block. In patients without PPI, a follow-up phone call was conducted at day 30 to collect the following clinical events: occurrence of syncope, hospital readmissions, subsequent PPI, and death. Data on pacemaker implantations and their follow-up were obtained prospectively and via remote monitoring 3 months after TAVI.

### Statistical analysis

Descriptive statistics were used to summarize baseline characteristics. Categorical variables were expressed as counts and percentages and compared using the χ^2^ test or Fisher’s exact test, as appropriate. Continuous variables were presented as means ± standard deviations and compared using the Student *t*-test or Mann–Whitney U test based on distribution assessed with the Shapiro–Wilk test. This study reports sex distribution and outcomes; however, it was not powered to detect sex-based interactions, and no consistent sex-specific differences were observed in secondary analyses. For key binary outcomes, exact 95% confidence intervals for proportions were calculated using the Clopper–Pearson method to reflect statistical uncertainty, particularly in the JR subgroup.

To balance the 2 groups of patients with JR and those without JR, and simulate a randomized comparison, we used the propensity score–matching approach to limit potential biases. We chose to use clinically relevant variables (age at implantation, sex, sinus rhythm before TAVI, hypertension, presence of LBBB or RBBB) that introduced imbalance between the 2 groups. Imbalance was judged by a standardized mean difference > 0.1. For each individual, the propensity score was estimated using a logistic regression model; the dependent variable was the JR group, and the independent variables were relevant clinical covariates. The Hosmer–Lemeshow test was used to verify the overall goodness of fit of the model. We used one-to-three nearest-neighbor matching without replacement, with a caliper width of 0.2 standard deviations of the logit of the propensity score, to limit matching to individuals with similar propensity scores. Graphical assessment of covariate balance was performed using a Love plot and the distribution of propensity scores before and after matching (Supplemental Figs. S1 and S2). Statistical analyses were performed using R software, version 4.3.0 and Stata v15 (StataCorp, College Station, TX), considering a 2-sided type I error rate of 5%.

## Results

Between December 1, 2023 and April 1, 2025, a total of 616 patients underwent TAVI at Clermont-Ferrand University Hospital, France. Among them, JR was identified in 26 patients, representing an incidence of 4.2%. For comparison, 78 patients without JR were propensity-matched with the JR group (Central Illustration).

**Central Illustration:**
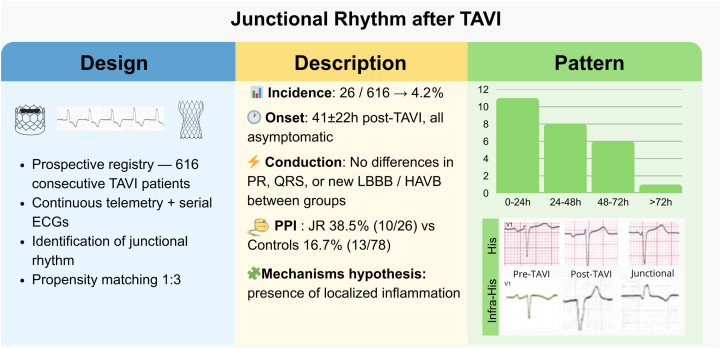
Junctional rhythm (JR) after transcatheter aortic valve implantation (TAVI): study design, clinical characteristics, and electrophysiological patterns. ECG, electrocardiogram. HAVB, high-grade atrioventricular block; LBBB, left bundle branch block; PPI, permanent pacemaker implantation.

### Clinical and procedural characteristics (Table 1)

Patients with JR had a mean age of 83.0 ± 5.3 years and were predominantly male (76.9%). Compared with patients without JR, there were no significant differences regarding cardiovascular risk factors or history of atrial fibrillation (15.4% vs 16.7%, *P* = 0.878). None of the matched patients had a PPI before TAVI or for persistent complete AVB occurring during the TAVI procedure. The logistic **Euro**pean **S**ystem for **C**ardiac **O**perative **R**isk **E**valuation EuroSCORE II did not differ significantly (12.8% ± 11.1% vs 14.2% ± 10.7%, *P* = 0.568). The left ventricular ejection fraction was similar (61.3% ± 9.4% vs 58.1% ± 9.1%, *P* = 0.144).

**Table 1:** Baseline clinical and procedural characteristics according to junctional rhythm status

Variable	No JR	JR	Total	*P*
**Baseline clinical characteristics**				
Male sex	61 (78.2)	20 (76.9)	81 (77.9)	0.891
Age at implantation, Y	82.5 ± 4.9	83.0 ± 5.3	82.6 ± 4.9	0.672
BMI, kg/m^2^	27.1 ± 4.9	26.4 ± 3.9	27.0 ± 4.7	0.456
NYHA class	2.4 ± 0.6	2.4 ± 0.6	2.4 ± 0.6	0.847
Diabetes mellitus	25 (32.1)	4 (15.4)	29 (27.9)	0.101
Hypertension	62 (79.5)	18 (69.2)	80 (76.9)	0.282
CAD history	28 (35.9)	12 (46.2)	40 (38.5)	0.352
Cardiac surgery history	6 (7.7)	2 (7.7)	8 (7.7)	1.000
Stroke history	5 (6.4)	4 (15.4)	9 (8.7)	0.159
Atrial fibrillation	13 (16.7)	4 (15.4)	17 (16.3)	0.878
Logistic EuroSCORE II, %	14.2 ± 10.7	12.8 ± 11.1	13.9 ± 10.8	0.568
Serum creatinine, μmol/L	112.9 ± 65.9	91.3 ± 26.3	107.5 ± 59.2	**0.019**
Creatinine clearance, mL/min	57.6 ± 28.1	59.7 ± 23.1	58.2 ± 26.9	0.704
Aortic annulus diameter, mm	23.9 ± 3.5	23.6 ± 2.3	23.8 ± 3.2	0.602
Mean gradient, mm Hg	43.7 ± 10.9	42.8 ± 12.2	43.5 ± 11.2	0.733
LVEF, %	58.1 ± 9.1	61.3 ± 9.4	58.9 ± 9.2	0.144
**Procedural and device characteristics**				
Valve type				
Balloon-expandable	30 (38.5)	12 (46.2)	42 (40.4)	0.489
Self-expanding	48 (61.5)	13 (50.0)	62 (59.6)	
Valve diameter, mm	27.3 ± 4.3	27.5 ± 2.7	27.4 ± 3.9	0.788
Balloon-expandable ≥ 29 mm	9 (30.0)	3 (25.0)	12 (28.6)	0,746
Self-expanding ≥ 29 mm	30 (62.5)	9 (64.3)	39 (62.9)	0,903
Valve/aortic annulus diameter ratio	1.1 ± 0.1	1.2 ± 0.1	1.2 ± 0.1	0.248
General anesthesia	19 (24.4)	4 (15.4)	23 (22.1)	0.340
Transfemoral approach	53 (67.9)	19 (73.1)	72 (69.2)	0.624
Subclavian approach	18 (23.1)	6 (23.1)	24 (23.1)	1.000
Residual mean gradient, mm Hg	8.2 ± 5.1	10.0 ± 6.6	8.6 ± 5.5	0.200
Pulmonary artery systolic pressure, mm Hg	29.9 ± 10.0	29.3 ± 7.8	29.8 ± 9.5	0.769
New permanent pacemaker implantation	13 (16.7)	10 (38.5)	23 (22.1)	**0.020**
Conduction disorder during hospitalization	8 (10.3)	3 (11.5)	11 (10.5)	0.478
Length of hospital stay, d	5.4 ± 2.6	5.8 ± 1.6	5.5 ± 2.4	0.346

Values are n (%) or mean ± standard deviation, unless otherwise indicated. Boldface indicates significance.

BMI : Body mass index. CAD : Coronary artery Disease; EuroSCORE II, **Euro**pean **S**ystem for **C**ardiac **O**perative **R**isk **E**valuation; JR, junctional rhythm; LVEF, left ventricular ejection fraction; NYHA, New York Heart Association.

Balloon-expandable valves were implanted in 46.2% of JR patients and 38.5% of controls (*P* = 0.489). Valve diameter (27.5 ± 2.7 vs 27.3 ± 4.3 mm, *P* = 0.788), valve/annulus ratio, and annulus dimensions did not differ significantly. The mean hospital stay was similar (5.8 ± 1.6 vs 5.4 ± 2.6 days, *P* = 0.346).

### JR characteristics

**Figure 1:**
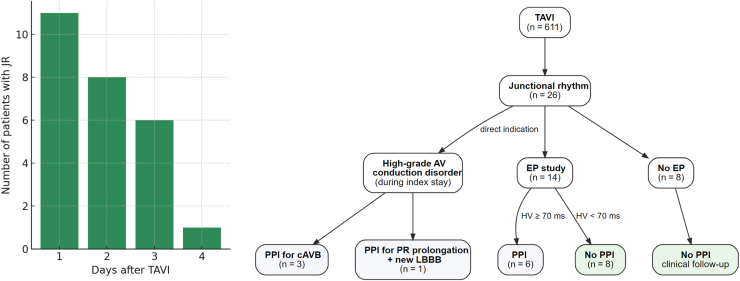
Diagram detailing (**left panel**) the timeline of junctional rhythm (JR) onset after transcatheter valve implantation (TAVI) and (**right panel**) evolution of patients with JR after TAVI procedure. AV, atrioventricular; cAVB, third-degree atrioventricular block; EP, electrophysiological; LBBB, left bundle branch block; PPI, permanent pacemaker implantation.

JRs were observed after aortic valve implantation, with a mean onset time of 41.1 ± 22.0 hours. The mean JR frequency was 71.2 ± 12.0 ms. JR was long-lasting (ie, ≥ 24 hours) in 2 patients (7.7%) (Figure 1). All patients were asymptomatic during JR rhythm. Dissociated atrial activity was identified in 57.7% of patients (n = 15), and 42.3% of patients (n = 11) had a retrograde P wave.

An electrophysiological study was performed in 53.8% of patients (n = 14). JRs were recorded in 1 patient. During this EPS, atrial pacing was performed to unmask atrioventricular conduction, and HV duration in JR was similar to the HV interval in sinus rhythm (54 ms) with a similar QRS during JR and sinus rhythm. During JR, His activation was concordant to a proximal activation of the His bundle.

**Figure 2:**
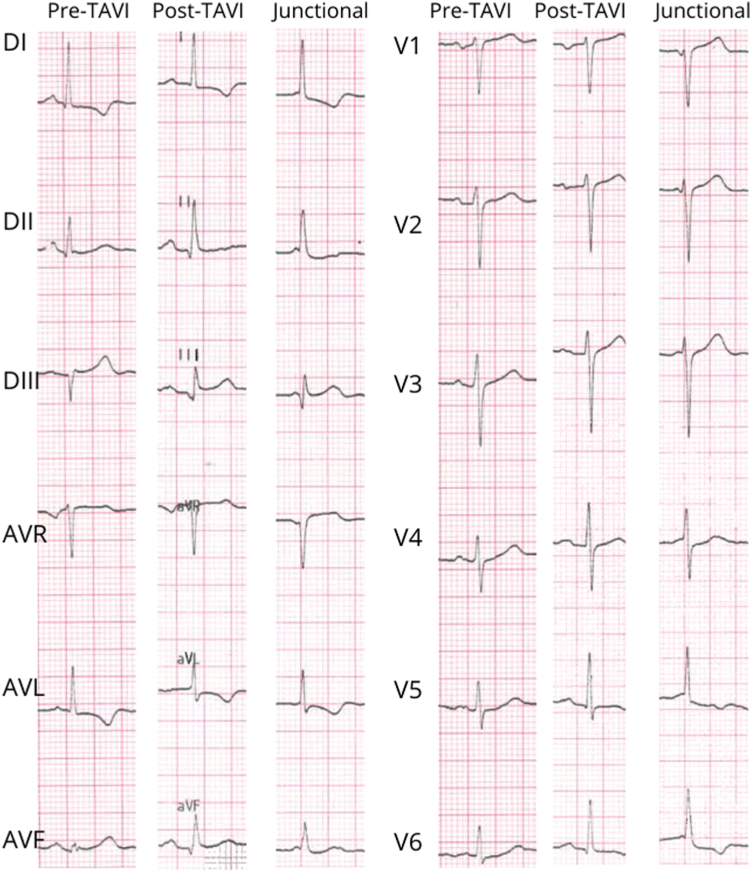
Hisian junctional rhythm (JR) following transcatheter aortic valve implantation (TAVI). Pre-TAVI: Baseline 12-lead electrocardiogram (ECG) showing normal sinus rhythm prior to TAVI. Post-TAVI: ECG on day 0 post-TAVI, comparable to the baseline tracing, with persistent sinus rhythm. Junctional: ECG performed 72 hours after TAVI revealing a JR with a ventricular rate of 65 beats per minute. See the first case description in Supplemental material: ***First case: hisian junctional rhythm***.

QRS morphology in JR was similar to supraventricular rhythm in 65.4% of patients (n = 17; see Case 1 in the Supplemental material, corresponding to Figure 2). Nine patients (34.6%) exhibited different QRS morphologies during sinus rhythm compared to JR (see Cases 2 and 3 in the Supplemental material, corresponding to Supplemental Figures S3 and S4). A left anterior pattern (ie, V1 positive, left deviation axis) was identified in 15.4% of patients (n = 4).

### Electrocardiographic characteristics (Table 2 and Fig. 2)

Before TAVI, patients with JR had heart rate, PR interval, and QRS duration comparable to those of controls. On day 1 post-TAVI, JR patients displayed a significantly lower mean heart rate (65 ± 10 vs 70 ± 15 beats per minute, *P* = 0.045), whereas the PR interval (213 ± 33 vs 206 ± 40 ms, *P* = 0.383) and QRS duration (125 ± 26 vs 130 ± 30 ms, *P* = 0.426) remained similar. All patients exhibited a sinus rhythm on the day-1 ECG, including those who had experienced JR during or immediately after TAVI. After TAVI, in the junctional group, 19.2% (n = 5) had new LBBB, and 53.8% (n = 14) had a QRS interval ≥ 100 ms. The proportion of patients with a QRS interval < 120 ms, new LBBB, or new RBBB did not differ significantly between groups.

**Table 2:** Electrocardiographic characteristics according to junctional rhythm (JR) status before and at day 1 after transcatheter aortic valve implantation (TAVI)

Variable	No JR	JR	Total	*P*
Pre-TAVI ECG				
Heart rate, bpm	70.8 ± 13.1	65.1 ± 14.7	69.3 ± 13.7	0.087
PR interval, ms	191.4 ± 36.6	193.4 ± 40.5	191.9 ± 37.4	0.825
QRS duration, ms	103.6 ± 23.8	99.7 ± 21.1	102.6 ± 23.1	0.437
QRS < 120 ms	66 (84.6)	19 (73.1)	85 (81.7)	0.187
Left bundle branch block	17 (21.8)	6 (23.1)	23 (22.1)	0.891
Right bundle branch block	2 (2.6)	1 (3.8)	3 (2.9)	0.735
ECG at day 1 post-TAVI				
Heart rate, bpm	70.3 ± 15.6	64.8 ± 10.5	68.9 ± 14.6	0.045
PR interval, ms	205.7 ± 39.7	213.3 ± 33.0	207.4 ± 38.3	0.383
QRS duration, ms	130.0 ± 29.9	125.0 ± 26.4	128.8 ± 29.0	0.426
QRS < 120 ms	35 (44.9)	11 (42.3)	46 (44.2)	0.820
Left bundle branch block	29 (37.2)	10 (38.5)	39 (37.5)	0.907
Right bundle branch block	3 (3.9)	1 (3.8)	4 (4.4)	0.854

Values are n (%) or mean ± standard deviation, unless otherwise indicated.

bpm, beats per minute; ECG, electrocardiogram.

### Conduction management and follow-up

PPI occurred in 38.5% of JR patients (95% confidence interval [CI]: 20.2%-59.4%) and 16.7% of controls (95% CI: 9.2%-26.8%). The distribution of pacemaker indications differed between groups (*P* = 0.025). The PPI incidence for complete AVB was similar (11.5% in JR patients [95% CI: 2.4%-30.2%] vs 10.3% in non-JR patients [95% CI: 4.5%-19.2%], *P* = 0.478). The PPI incidence was more frequent in the JR group for non–high-grade conduction indications at 26.9% (95% CI: 11.6%-47.8%), compared with non-JR patients at 6.4% (95% CI: 2.1%-14.3%), *P* = 0.008.

In the JR group, the pacemaker indication in patients without high-grade conduction disturbance was prolonged HV interval ≥ 70 ms + LBBB in 3 patients (23%), HV interval ≥ 70 ms + widened QRS interval in 2 patients, prolonged HV+ normal QRS in 1 patient, and combined PR prolongation + new onset LBBB in 1 patient. Patients undergoing PPI had prolonged QRS duration (127.3 ± 20.1 vs 104.8 ± 24.3 ms, *P* = 0.013) and prolonged QTc interval. No significant differences were found regarding junctional heart rate, QRS morphology or axis, atrial rate, or VA interval (Table 3).

**Table 3:** Pacemaker implantation according to electrocardiogram characteristics in junctional rhythm

Characteristic	No PPI (n = 16)	PPI (n = 10)	Total (n = 26)	*P*
Junctional HR, bpm	71.4 ± 13.2	70.9 ± 10.0	71.2 ± 12.0	0.8
QRS interval (ms)	104.8 ± 24.3	127.3 ± 20.5	112.9 ± 25.1	**0.013**
QRS morphology				0.22
Normal	11 (68.5)	3 (30.0)	14 (53.8)	
LBBB	2 (12.5)	2 (20.0)	4 (15.4)	
RBBB	2 (12.5)	1 (10.0)	3 (11.5)	
RBBB + LPFB	1 (6.3)	2 (20.0)	3 (11.5)	
RBBB + LAFB	0 (0.0)	1 (10. 0)	1 (3.9)	
NICD + LAFB	0 (0.0)	1 (10.0)	1 (3.9)	
Axis				0.13
Normal	12 (75.0)	4 (40.0)	16 (61.5)	
Left	3 (18.8)	5 (50.0)	8 (30.8)	
Right	1 (6.3)	1 (10.0)	2 (7.7)	
QTc, ms	414.9 ± 17.2	450.4 ± 25.7	427.7 ± 26.6	**< 0.001**
Atrial rate, ms	946.7 ± 170.9	880.0 ± 54.2	926.2 ± 145.7	0.43
VA interval, ms	145.0 ± 169.2	75.0 ± 150.0	110.0 ± 152.7	0.51
CRP, mg/L	37.3 ± 24.3	54.1 ± 28.0	44.0 ± 26.5	0.23

Values are n (%) or mean ± standard deviation, unless otherwise indicated. Boldface indicates significance.

Bpm, beats per minute; CRP, C-reactive protein; HR, heart rhythm; LAFB, left anterior fascicular block; LBBB, left bundle branch block; LPFB, left posterior fascicular block; NICD, nonspecific intraventricular conduction delay; PPI, permanent pacemaker implantation; RBBB, right bundle branch block; VA, ventricul to atria.

At 30 days following TAVI, none of the patients with JR who had not received a PPI experienced syncope, were rehospitalized, or died. At 3 months, of the 10 patients who were implanted with a PPI, 5 patients exhibited a ventricular pacing burden of 0%. One patient demonstrated 100% ventricular pacing; this patient had received left bundle branch area pacing due to left ventricular dysfunction in the context of LBBB and HV ≥ 70 ms. In the remaining 4 patients, ventricular pacing burden at 3 months was 3% (2 patients), 41% (1 patient), and 50% (1 patient), respectively. Analysis of pacemaker holter memory revealed episodes of sustained supraventricular tachycardia in 3 of the 10 patients. Among the 4 patients implanted with a Microport device (MicroPort Medical Co. Ltd., Shanghai, China) programmed in an atrial-based pacing mode (AAI) with automatic conversion to dual-chamber pacing (DDD) using the SafeR algorithm designed to minimize unnecessary ventricular pacing, one showed episodes of complete AVB recorded in the memory at the 3-month follow-up (Supplemental Table S1). No significant difference occurred in in-hospital or 3-month mortality, with 2 deaths (noncardiovascular) reported in the non-JR group.

## Discussion

This study provides insights into the characterization of JR in the context of TAVI. JR is an underrecognized and consistently asymptomatic phenomenon after TAVI. Its apparent low incidence likely reflects underdetection, as it is transient, often subtle on ECG, and does not trigger telemetry alarms.

When compared to a matched cohort of patients without JR, no significant differences were found regarding baseline characteristics, procedural parameters, or post-TAVI complications. No identifiable clinical, electrocardiographic, or procedural risk factors were associated with the occurrence of JR. An important finding was that JR was observed both in patients with preexisting conduction disorders and in those with normal baseline atrioventricular conduction.

Two distinct patterns were observed. In some patients, the escape rhythm displayed a QRS morphology similar to the conducted QRS during sinus rhythm, which is consistent with a high-junctional origin (atrioventricular nodal or proximal His). In others, the escape rhythm exhibited a different, wider QRS morphology with a bundle-branch block pattern. Although this latter pattern may suggest involvement of the His–Purkinje system and a more distal origin, the precise level (intra-Hisian vs infra-Hisian, or even distal to the His bifurcation) cannot be established without His bundle recordings obtained during the rhythm.

Mechanistically, one may hypothesize that in a patient developing LBBB after TAVR due to longitudinal dissociation of the His bundle (ie, selective injury to left-sided His fibers), an accelerated escape rhythm could arise just distal to the intra-His lesion yet proximal to the bifurcation, thereby producing an RBBB-pattern escape rhythm. Such proof remains exceptional; a representative example is the case reported by Sekihara et al., in which intracardiac recordings supported a persistent accelerated infra-Hisian escape rhythm masking infranodal AVB after TAVI.^15^ In our cohort, we did not obtain systematic His bundle recordings during wide-QRS escape rhythms, precluding any definitive statement regarding an intra-Hisian or infra-Hisian origin.

This topographic heterogeneity, combined with the transient and self-limiting course of JR, suggests a mechanism that is unlikely to be purely mechanical. A plausible hypothesis is the presence of localized inflammation affecting the atrioventricular conduction system after valve deployment. Although post-TAVI inflammatory conduction disturbances are not well documented, such a mechanism aligns with observations from other clinical contexts in which JR occurs, including cardiac surgery and inflammatory cardiomyopathies.

Our management of JR after TAVI does not rely on the rhythm itself, but rather on the associated conduction abnormalities, whether preexisting or newly developed. The rate of PPI in patients with JR was not comparable to that of patients without JR and was not always driven by established indications. Numerous electrophysiological studies were performed in patients with JR despite the absence of formal indications, as no conduction disturbances were documented.^11^^,^^16^ In some cases, this led to pacemaker implantation based solely on an HV interval ≥ 70 ms, even for patients with a QRS interval < 120 ms and no LBBB. At the time, we tended to consider JR as a potential aggravating factor for conduction.

There is a risk of identifying HV prolongation, which is common in severe aortic stenosis and may inappropriately influence clinical management. An exception could be in cases in which baseline rhythm is not observable due to persistent JR, in which case atrial pacing may be justified to unmask the native QRS complex and help stratify the patient’s conduction risk. Therefore, in the absence of persistent or progressive conduction block, JR alone does not seem to justify prophylactic pacemaker implantation.

**Figure 3:**
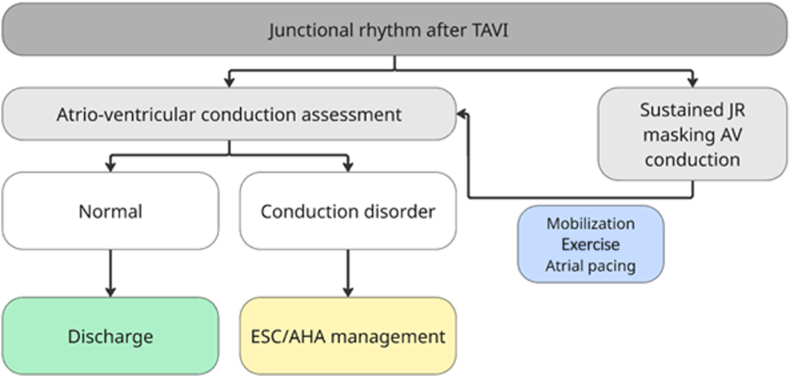
Practical approach to junctional rhythm (JR) after transcatheter aortic valve implantation (TAVI). AHA, American Heart Association; AV, atrioventricular; ESC, European Society of Cardiology

The 2021 European Society of Cardiology guidelines on valvular heart disease do not provide specific recommendations regarding JR after TAVI. Current guidance on conduction disturbances mainly addresses new-onset LBBB, PR/QRS prolongation, or high-grade AVB, with an EPS or prolonged monitoring in selected cases.^17^ From a practical standpoint, 3 scenarios can be distinguished. First, when JR is sustained but occurs in the absence of any conduction disturbance, it should simply be ignored and does not justify additional interventions or prolonged hospitalization. Second, when JR coexists with conduction abnormalities, such as high-grade AVB, PR prolongation, or bundle branch block, the management should follow the current European or North American recommendations for post-TAVI conduction disturbances, irrespective of the JR itself. Finally, when JR masks the electrocardiographic assessment of conduction (making it difficult to evaluate the PR interval or the QRS duration), strategies such as restoring sinus rhythm predominance (mobilization, exercise testing, or atrial pacing during EPS) or waiting for the disappearance of JR are required to make an informed decision (Fig. 3).

JR should be actively recognized during post-TAVI monitoring, as it may mimic more severe conduction disturbances, such as alternating bundle branch block, complete AVB, or atrial fibrillation when atrial activity is poorly visible. Early and accurate identification is essential to improve patient management and avoid unnecessary interventions. Such misreadings carry the risk of overestimating conduction system instability, potentially leading to unwarranted pacemaker implantation. Once it is correctly identified, isolated JR in the absence of progressive or high-grade conduction abnormalities does not in itself justify further electrophysiological evaluation or prophylactic pacemaker implantation. For this reason, it is crucial for the cardiology community, particularly those involved in post-TAVI monitoring, to develop a familiarity with the electrocardiographic features of JRs, to understand their benign and transient nature, and to interpret them critically within the broader clinical context.

### Limitations

This study was conducted as a single-centre cohort, which may limit the external validity and generalizability of our findings. Given the asymptomatic nature of JRs and their poor detectability on continuous telemetry, many episodes likely were missed. Detection either relied on proactive ECG review, as JR does not trigger telemetry alerts, or occurred incidentally on routine ECGs. As a result, the true incidence of JR post-TAVI is underestimated, and our reported rate may not reflect its actual prevalence.

Regarding clinical management, the absence of a standardized protocol for JR led, at times, to the presumption of conduction system instability, which may have influenced decisions toward earlier pacemaker implantation in this subgroup. PPI reflects both objective conduction deterioration and physician-driven risk mitigation strategies, introducing treatment bias. However, analysis of pacemaker interrogation data did not support sustained conduction abnormalities in these patients. Notably, none of the patients were pacemaker-dependent during follow-up. In the subset of patients implanted after an EPS with a pacemaker device, which allow detailed event logging, including episodes of complete AVB (4 of 8), only one patient of 4 experienced an episode of third-degree AVB during follow-up after discharge. These findings support the notion that the rhythm itself did not indicate latent advanced conduction disease. We did not systematically quantify implantation depth or membranous septum length, which are recognized predictors of post-TAVI conduction disturbances. We used appropriate statistical methods (χ^2^ and/or Fisher’s exact tests as appropriate); nonsignificant *P*-values should not be interpreted as evidence of no association, but rather as reflecting limited statistical power and imprecision of effect estimates.

## Conclusion

In this prospective single-centre study, JR after TAVI appeared to be an infrequent, transient, and clinically silent phenomenon. Although pacemaker implantation was more common among JR patients, this difference was not associated with a higher incidence of documented high-grade AVB and was driven largely by discretionary electrophysiological decisions. Given the limited number of JR patients and the resulting imprecision of effect estimates, these findings should be considered exploratory. Larger multicentre studies are warranted to confirm the clinical significance of JR after TAVI.

## Data Availability

The data underlying this article will be shared on reasonable request to the corresponding author.

## Ethics Statement

The study was approved by the local ethics committee (CPP Sud Est VI, 2023/ 24.03051.000665). The research reported in this paper adhered to the CONSORT guidelines.

## Patient Consent

The authors confirm that patient consent forms have been obtained for this article.

## Funding Sources

The authors have no funding sources to declare.

## Disclosures

The authors have no conflicts of interest to disclose.

## Footnotes

To access the supplementary material accompanying this article, visit *CJC Open* at https://www.cjcopen.ca/ and at https://doi.org/10.1016/j.cjco.2026.03.005
