# Supplementary material for: Junctional Rhythms After Transcatheter Aortic Valve Implantation: Incidence, Temporal Patterns, and Clinical Outcomes
Source: CJC Open. 2026 Mar 18;8(7):842–50. doi: 10.1016/j.cjco.2026.03.005 (PMC13386741; doi:10.1016/j.cjco.2026.03.005)
Supplement: Supplementary Material [file mmc1.docx]

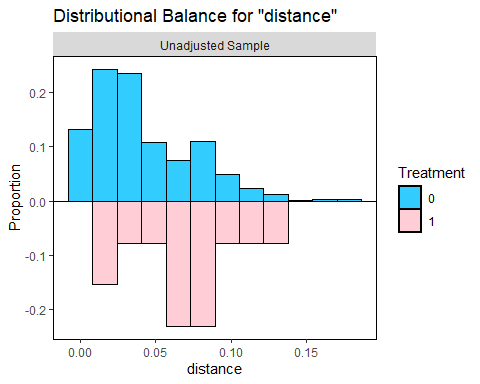

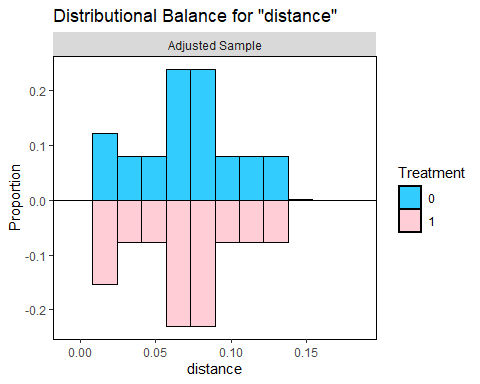


Supplemental Figure S1. Distributional balance for distance before and after propensity score matching. Kernel density plots of the estimated propensity scores in patients with and without junctional rhythm before and after matching.


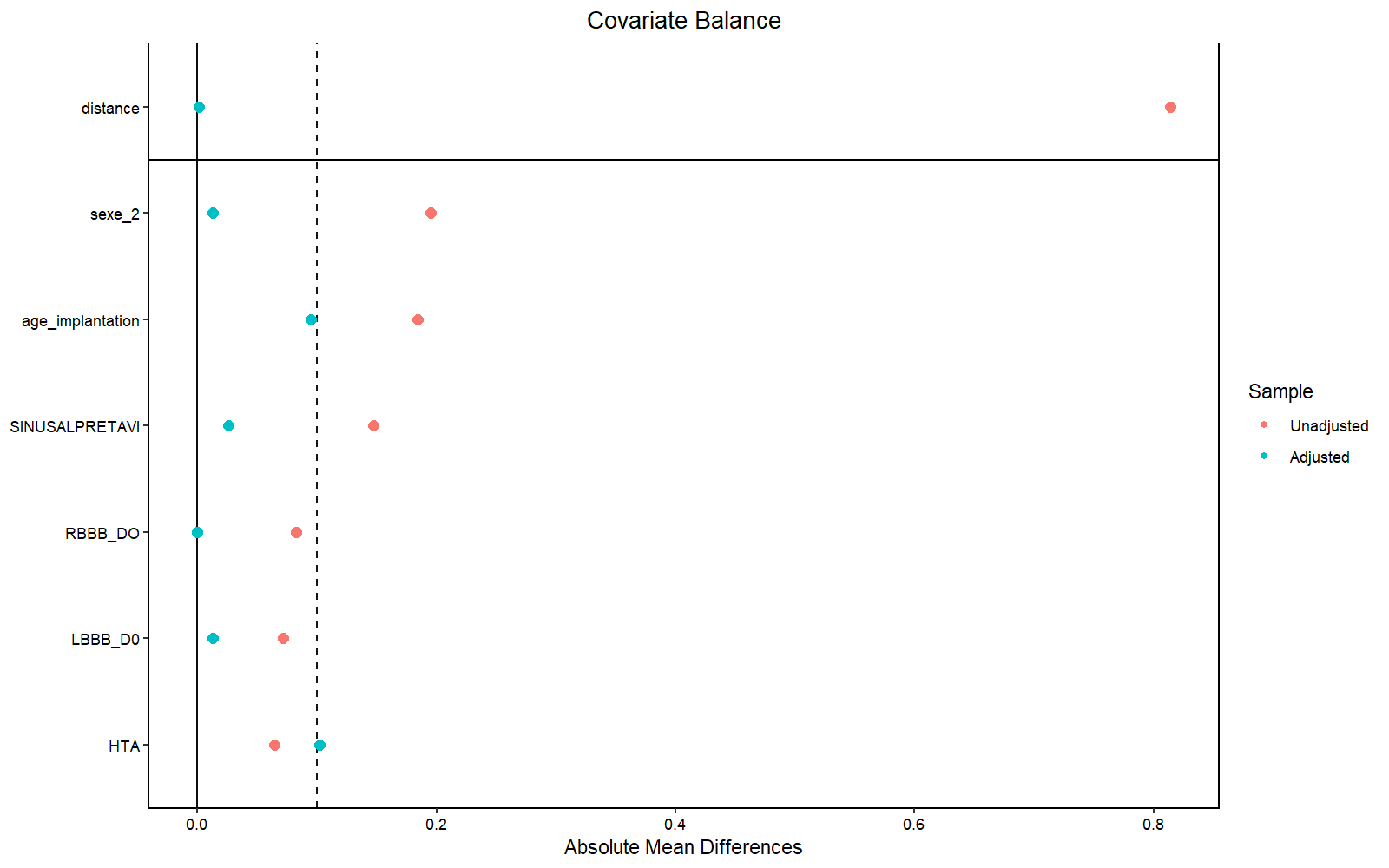

Supplemental Figure S2. Standardized mean differences before and after propensity score matching (Love plot). Each dot represents the standardized mean difference for one covariate before and after matching. The vertical dashed line indicates the 0.1 threshold for acceptable balance.

***First case: hisian junctional rhythm (Figure 2)***

The pre-TAVI ECG showed a sinus rhythm with a PR interval of 152 ms and a normal QRS morphology with a QRS duration of 94 ms. Post-TAVI, the rhythm remained sinus, with a modest PR prolongation to 205 ms and a similar QRS duration. A JR was observed, with an identical QRS to that of the post-TAVI sinus rhythm. This superimposable ECG appearance suggested a purely hisian escape rhythm. Electrophysiological study confirmed this hypothesis, with an HV interval measured at 60 ms. In the absence of severe conduction abnormalities, no indication for PPI was retained. At 30 days post-TAVI, the patient had no episodes of syncope or presyncope and did not require rehospitalization.

***Second case :* Accelerated escape rhythm with RBBB morphology and de novo-LBBB after TAVI*.***

The pre-TAVI electrocardiogram showed a sinus rhythm with a PR interval of 180 ms and narrow QRS complexes measuring 92 ms.

At 48 hours post-implantation, the patient developed a JR with a RBBB and a QRS duration of 135 ms. A remarkable electrocardiographic phenomenon was observed: the presence of a fusion complex on the JR ECG, which reproduced the narrow QRS morphology seen preoperatively. This finding unmasked an LBBB, as the competition between the junctional escape with RBBB morphology and sinus conduction resulted in a perfect revealing fusion.

Electrophysiological study revealed a prolonged HV interval of 94 ms, establishing the indication for permanent pacing. A left bundle branch pacing device was implanted in the setting of impaired left ventricular function.


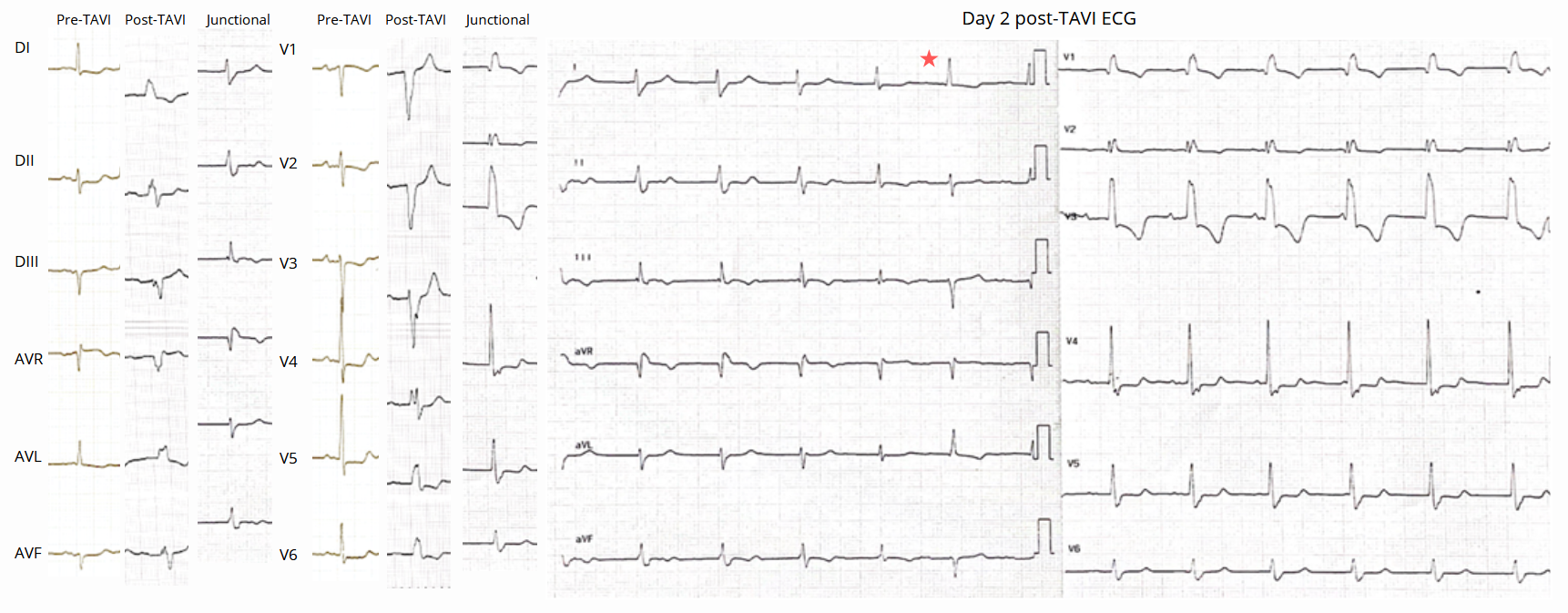


**Supplemental Figure S3. Accelerated escape rhythm with RBBB morphology 48 hours after transcatheter aortic valve implantation.** **Pre-TAVI :** Baseline ECG showing sinus rhythm with narrow QRS complexes and bigeminal premature ventricular contractions. **Post-TAVI :** Electrocardiogram recorded in sinus rhythm 48 hours after TAVI showing a LBBB. **Junctional :** ECG recorded 48 hours post-TAVI showing JR with a RBBB morphology. Lower panel : Electrocardiogram in JR showing a fusion complex (star) with morphology identical to the pre-TAVI ECG, unmasking an underlying LBBB.

***Third case : Wide QRS junctional rhythm with*** left anterior ***morphology***

The pre-TAVI ECG demonstrated sinus rhythm with a PR interval of 164 ms and a LBBB (QRS duration 144 ms). After TAVI, the ECG still showed a LBBB of the same duration, with a stable PR interval of 170 ms.

On day 3 post-procedure, the patient developed a JR at 100 bpm, associated with a RBBB (QRS 120 ms) and left axis deviation. An electrophysiological study revealed an HV interval of 54 ms, therefore pacemaker implantation was not indicated. At 30-day follow-up, the patient remained asymptomatic, without syncope, rehospitalization, or heart failure.


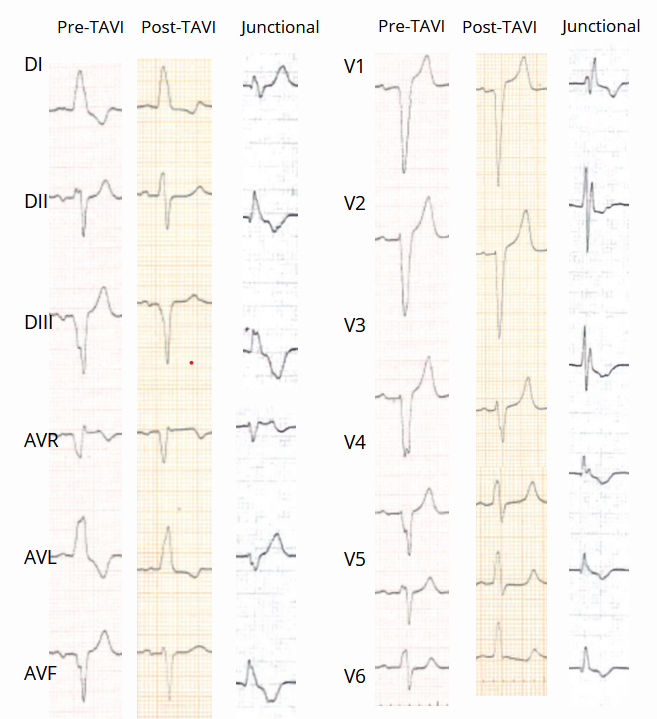


**Supplemental Figure S4.** **Accelerated escape rhythm with** left anterior **morphology 3 days after transcatheter aortic valve implantation (TAVI). Pre-TAVI:** sinus rhythm with LBBB (QRS duration 144 ms). **Post-TAVI**: similar ECG findings with stable PR interval and QRS duration. **Junctional**: JR at 100 bpm with RBBB and left axis deviation

|  | Case 1 | Case 2 | Case 3 | Case 4 | Case 5 | Case 6 | Case 7 | Case 8 | Case 9 | Case 10 |
| --- | --- | --- | --- | --- | --- | --- | --- | --- | --- | --- |
| Type of valve | Balloon-expandable | Self-expandable | Balloon-expandable | Self-expandable | Balloon-expandable | Self-expandable | Self-expandable | Balloon-expandable | Self-expandable | Self-expandable |
| Beta-blocker use | Yes | Yes | Yes | No | Yes | No | Yes | No | No | Yes |
| Baseline PR interval (ms) | 216 | 212 | 170 | 190 | 220 | 160 | 180 | 240 | 224 | 186 |
| Baseline QRS interval (ms) | 138 | 86 | 125 | 120 | 90 | 94 | 92 | 90 | 94 | 132 |
| Baseline QRS morphology | LBBB | Normal | NICD + LAFB | RBBB + LAFB | Normal | Normal | Normal | Normal | Normal | NICD |
| Post-operative PR interval (ms) | 200 | junctional rhythm | 192 | 210 | 230 | 190 | 180 | 220 | 247 | 169 |
| Post-operative QRS interval (ms) | 140 | junctional rhythm | 139 | 140 | 110 | 145 | 127 | 140 | 173 | 136 |
| Post-operative QRS morphology | LBBB | junctional rhythm | NICD + LAFB | RBBB + LAFB | Normal | LBBB | NICD + LAFB | LBBB | LBBB | NICD |
| Rate of junctional rhythm (bpm) | 61 | 72 | 64 | 59 | 66 | 92 | 76 | 68 | 75 | 73 |
| Junctional QRS interval (ms) | 135 | 127 | 125 | 130 | 100 | 102 | 135 | 140 | 170 | 122 |
| Junctional QRS morphology | LBBB | RBBB | NICD + LAFB | RBBB + LAFB | Normal | Normal | RBBB | LBBB | RBBB + LPFB | LBBB |
| AV dissociation | No | Yes | No | No | No | Yes | Yes | Yes | No | Yes |
| Post-procedural day of detection | 3 | 0 | 2 | 3 | 2 | 2 | 2 | 1 | 1 | 1 |
| EP study | Yes | No | Yes | No | Yes | No | Yes | No | Yes | Yes |
| HV (ms) | 76 | - | 78 | - | 76 | - | 94 | - | 88 | 100 |
| AV block during hospitalization | No | Yes | No | Yes | No | No | No | No | No | No |
| Junctional stimulation rate at 3 months (%) | 0 | 3 | 0 | 41 | 0 | 3 | 100 | 0 | 50 | 0 |
| AV block on holter at 3 months | - | No | - | Yes | No | - | - | - | - | Yes |

Supplemental Table S1. Characteristics and outcomes of patients who developed junctional rhythm and implanted with PPI
